# Development of Red Exciplex for Efficient OLEDs by Employing a Phosphor as a Component

**DOI:** 10.3389/fchem.2019.00016

**Published:** 2019-01-31

**Authors:** Ming Zhang, Kai Wang, Cai-Jun Zheng, De-Qi Wang, Yi-Zhong Shi, Hui Lin, Si-Lu Tao, Xing Li, Xiao-Hong Zhang

**Affiliations:** ^1^School of Optoelectronic Science and Engineering, University of Electronic Science and Technology of China, Chengdu, China; ^2^Institute of Functional Nano & Soft Materials (FUNSOM) and Jiangsu Key Laboratory for Carbon-Based Functional Materials & Devices, Soochow University, Suzhou, China

**Keywords:** exciplex, thermally activated delayed fluorescence, phosphor, spin-orbit coupling, non-radiative decay

## Abstract

Exciplexes are ideal candidates as effective thermally activated delayed fluorescence (TADF) emitters. However, efficient orange and red TADF exciplexes have been reported seldomly, because their significant non-radiative (NR) decay of excited states lead to unavoidable energy loss. Herein, we propose a novel strategy to construct efficient red TADF exciplexes by introducing phosphor as one component. Due to the strong spin–orbit coupling of heavy metal (e.g., Ir, Pt, et al.) ion cores, the NR decays will be evidently decreased for both singlet and triplet excitons, reducing the undesired exciton waste. Moreover, compared with the conventional exciplexes, phosphorescence plays an important role for such novel exciplexes, further improving the exciton utilization. Based on this strategy, we fabricated a red exciplex containing 1,3,5-triazine-2,4,6-triyl)tris(benzene-3,1-diyl)tris(diphenylphosphine oxide) (PO-T2T) and tris(2-phenylpyridine) iridium(III) (Ir(ppy)_3_) as components and realize a red emission with a peak at 604 nm, a CIE coordinate of (0.55, 0.44), and a high maximum external quantum efficiency of 5% in organic light-emitting device. This efficiency is 2.6 times higher than that of the device based on the conventional red exciplex emitter, proving the superiority of our novel strategy to construct TADF exciplexes with phosphors.

## Introduction

**Figure 1:**
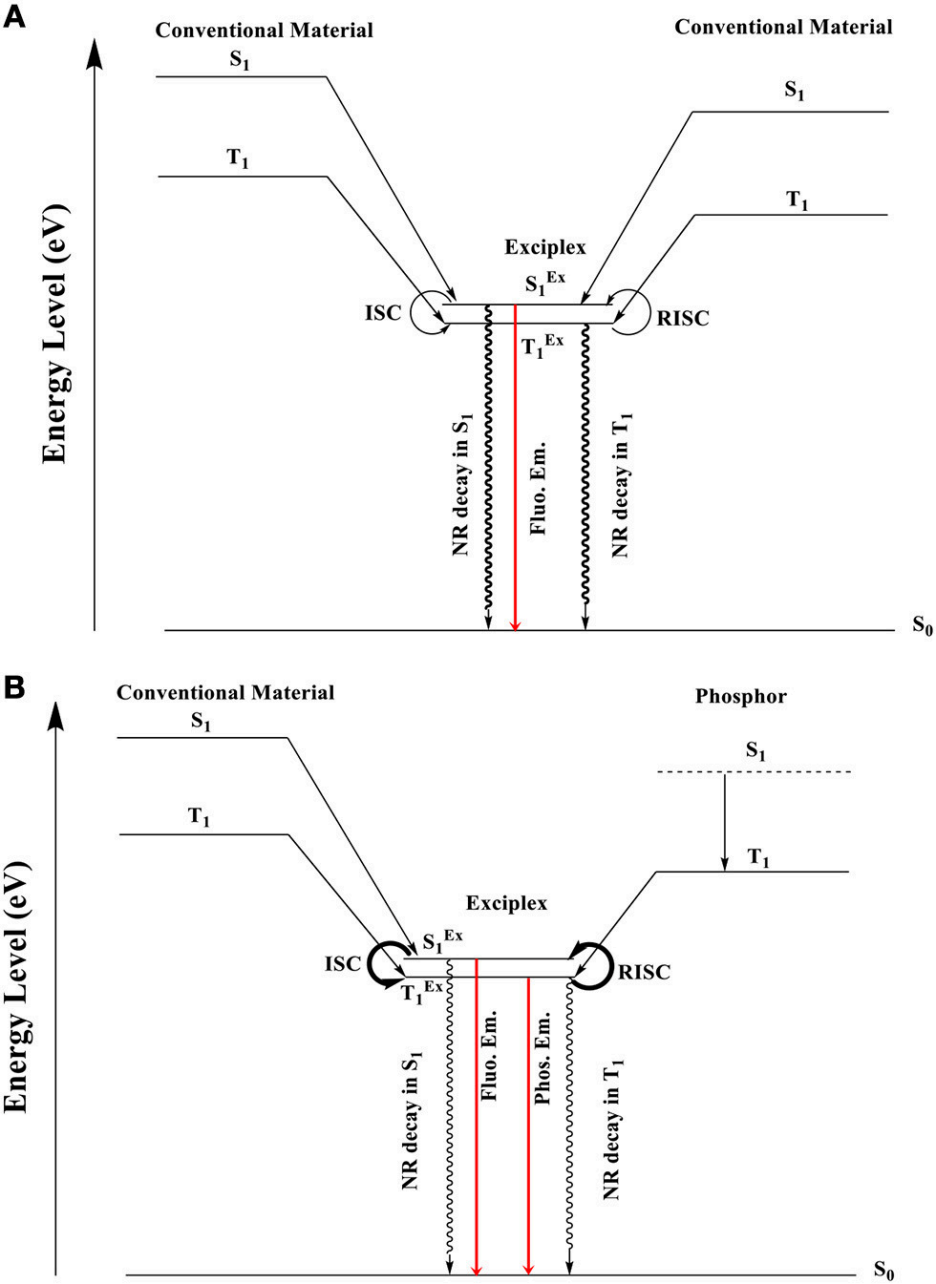
Energy transfer diagrams of the exciplex systems employing **(A)** two conventional fluorescent materials and **(B)** one phosphor and one conventional fluorescent material. S_1_, T_1_, and S_0_ are the lowest singlet excited state, lowest triplet excited state and ground state, respectively.

Organic light-emitting devices (OLEDs) based on thermally activated delayed fluorescence (TADF) emitters are considered the new generation of OLEDs (Adachi, 2014) and have drawn great attention in recent years (Goushi et al., 2012; Uoyama et al., 2012; Zhang et al., 2014b, 2016; Ban et al., 2015; Hirata et al., 2015; Liu M. et al., 2015, 2017; Liu W. et al., 2015; Chen et al., 2016, 2017; Gómez-Bombarelli et al., 2016; Li et al., 2016, 2017, 2018; Xie et al., 2016; Miwa et al., 2017; Moon et al., 2017; Shiu et al., 2017; Wang K. et al., 2017a; Yang et al., 2017). The TADF emitters can utilize both singlet and triplet excitons for emission by up-converting non-radiative (NR) triplet excitons to radiative singlet excitons via an efficient reverse intersystem crossing (RISC) process from lowest triplet state (T_1_) to lowest singlet state (S_1_) (Uoyama et al., 2012). Theoretically, an effective RISC process requires that the TADF emitter possesses an extremely small singlet-triplet energy splitting (Δ*E*_ST_) between S_1_ and T_1_ (Uoyama et al., 2012), which needs to isolate its highest occupied molecular orbital (HOMO) and lowest unoccupied molecular orbital (LUMO) (Chen et al., 2016; Wang D. Q. et al., 2017; Wang et al., 2017b). Clearly, exciplexes are among the ideal candidates as effective TADF emitters, because they meet the requirements mentioned above naturally (Liu et al., 2015a,b, 2016). Formed via intermolecular charge-transfer (CT) transition, the HOMOs and LUMOs of exciplexes are independently located on the electron-donor (D) and electron-acceptor (A) component molecules respectively, possessing extremely small overlaps. Thus, exciplex systems present extremely small energy gaps between their own ^1^CT and ^3^CT (<0.1 eV). By using high-T_1_ component molecules to avoid the triplet excitons loss, exciplexes commonly exhibit TADF characteristics. In the past few years, great progress has been made for exciplex-based TADF OLEDs (Goushi et al., 2012; Li et al., 2014; Liu et al., 2015b,c, 2016; Zhang et al., 2017; Shi et al., 2018). In 2012, Adachi and co-workers firstly reported a yellow-green TADF exciplex, and theoretically proved its great potential (Goushi et al., 2012). In 2015, our group developed a novel strategy to predict and design efficient exciplex with TADF characteristics by using the HOMO and LUMO energy levels of constituting molecules (Liu et al., 2015a). And in 2016, our group further reported highly efficient exciplex-based TADF OLED with an high external quantum efficiency (EQE) of 17.8% (Liu et al., 2016). However, nearly all the currently reported exciplex TADF emitters are limited in short-wavelength (i.e., blue, green, and yellow) emission (Goushi et al., 2012; Li et al., 2014; Liu et al., 2015b,c, 2016; Zhang et al., 2017; Shi et al., 2018). Efficient TADF exciplexes with long-wavelength (i.e., orange and red) emission have rarely been reported (Data et al., 2016). Figure 1A illustrates the exciton transfer processes in conventional TADF exciplexes. Both the constituting molecules should have higher S_1_ and T_1_ energy levels than that of exciplex, ensuring all excitons can be harvested on S_1_ and T_1_ states of exciplex (Liu et al., 2015a) Therefore, the energy loss of the exciplex-based TADF OLEDs should be mainly caused by the NR decay of both exciplex S_1_ and T_1_ states. According to photophysical theory, the rate constants of the NR decays are exponentially magnified with the decreased bandgap energy level (Zhang et al., 2014a). For TADF exciplexes with short-wavelength emission, the NR decays of S_1_ and T_1_ states are almost neglectful compared with the emission process of S_1_ state and RISC process of T_1_ state, realizing high exciton utilization in the OLEDs (Liu et al., 2016). While for common orange and red TADF exciplexes, the NR decays of excited states become significant due to narrow bandgaps, leading to unavoidable energy loss. Therefore, efficient orange and red TADF exciplexes are hard to be achieved by these common exciplex systems and become the bottleneck of exciplexes development.

In this study, we proposed a novel strategy to construct efficient red exciplexes by introducing a phosphor as one component. As we known, phosphors are generally heavy metal complexes. The spin–orbit coupling (SOC) of heavy metal (Ir, Pt, et al.) ion cores will significantly enhance the energy transfer processes between singlet and triplet states [intersystem crossing process (ISC) from S_1_ to T_1_ and phosphorescent decay from T_1_ to S_0_], allowing heavy metal complexes to achieve phosphorescence efficiently (Baldo et al., 1998; Liu X. et al., 2015; Liu B. et al., 2017). Correspondingly, in new type exciplexes, the decay routes of excited exciplexes will be also affected by the heavy metal phosphor cores. The ISC process from ${\text{S}}_{1}^{\text{EX}}$ to ${\text{T}}_{1}^{\text{EX}}$, RISC process from ${\text{T}}_{1}^{\text{EX}}$ to ${\text{S}}_{1}^{\text{EX}}$ and phosphorescent decay from ${\text{T}}_{1}^{\text{EX}}$ to ${\text{T}}_{0}^{\text{EX}}$ can be significantly enhanced by the SOC effect. Thus, the proportions of the NR decays will be accordingly decreased for ${\text{T}}_{1}^{\text{EX}}$ and ${\text{T}}_{1}^{\text{EX}}$ states, reducing the undesired exciton waste. Moreover, compared with the conventional exciplexes, phosphorescence may play an important role on such new type of exciplexes with phosphor (Cherpak et al., 2015) (Figure 1B), further improving the exciton utilization. Based on this novel strategy, we fabricated a red exciplex containing 1,3,5-triazine-2,4,6-triyl)tris(benzene-3,1-diyl)tris(diphenylphosphine oxide) (PO-T2T) and tris(2-phenylpyridine) iridium(III) (Ir(ppy)_3_) as components, which exhibits typical TADF characteristic with an extremely small Δ*E*_ST_ of 0.026 eV. In device, PO-T2T:Ir(ppy)_3_ exciplex shows a red emission with a peak at 604 nm and a CIE coordinate of (0.55, 0.44), and realizes a high maximum external quantum efficiency (EQE) of 5%. As a comparison, the conventional TADF exciplex consisting of PO-T2T and 1,3-di(10H-phenoxazin-10-yl)benzene (13PXZB) only achieves a maximum EQE of 1.9% in device. These results not only demonstrate exciplexes can also be formed by using a phosphor component, but also indicate that the NR decays of excited states can be significantly suppressed for phosphor-based exciplexes by the SOC effect of heavy metal ion core from phosphor. Although noble metal Ir is contained in our system, we believe efficient but low-cost phosphor-based exciplex emitters can be developed by using other cheap heave metal-based phosphors, like Cu, et al.

## Results and Discussion

**Figure 2:**
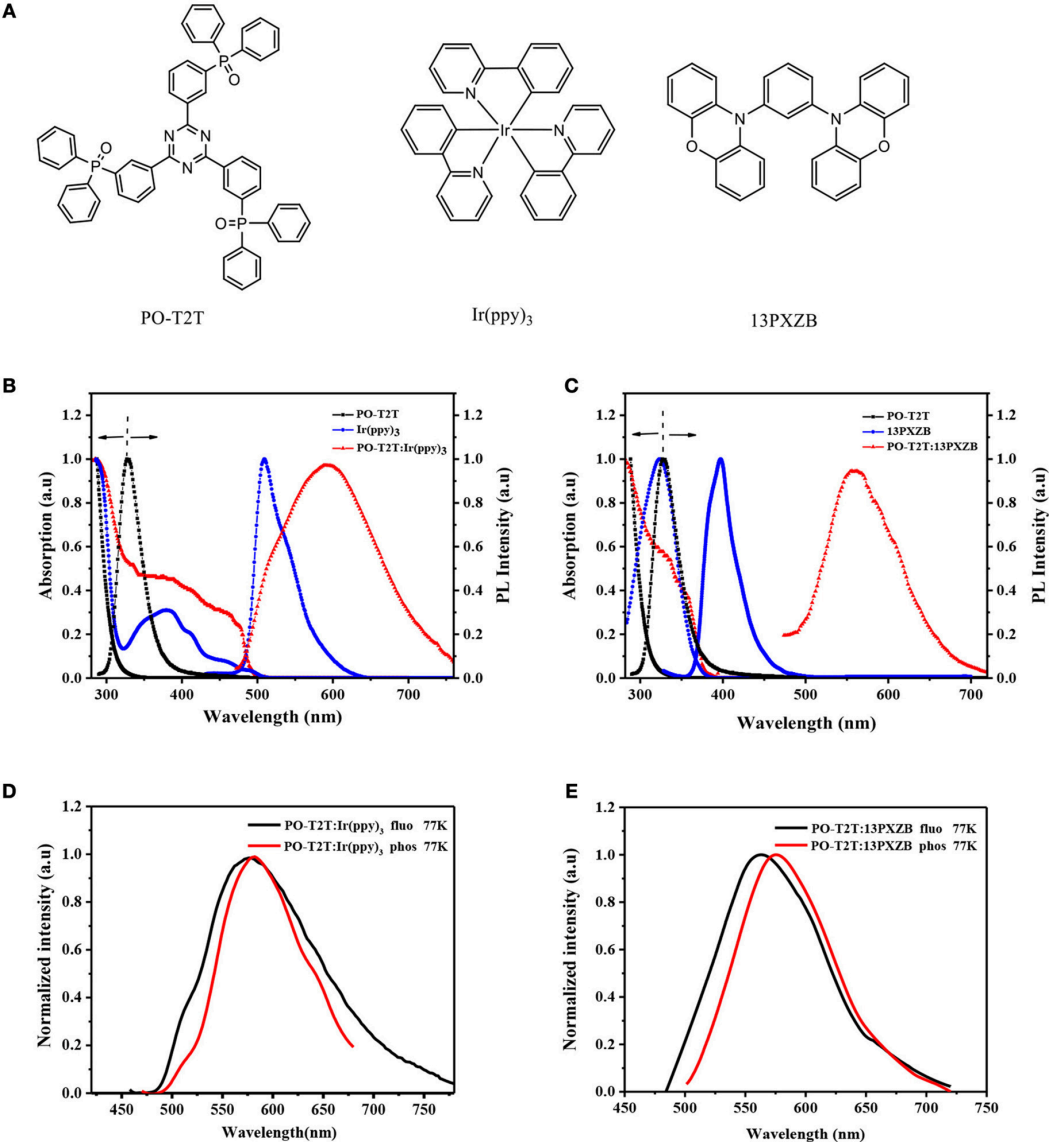
**(A)** Chemical structures of PO-T2T, Ir(ppy)_3_ and 13PXZB; **(B)** Absorption and PL spectra of PO-T2T, Ir(ppy)_3_ in toluene solution and POT2T:Ir(ppy)_3_ in solid thin film; **(C)** Absorption and PL spectra of PO-T2T, 13PXZB in toluene solution and POT2T:13PXZB in solid thin film; **(D)** the fluorescence and phosphorescence spectra of PO-T2T: 8 wt% Ir(ppy)_3_ mixed film and **(E)** PO-T2T: 40 wt% 13PXZB mixed film.

Figure 2A illustrates the molecular structures of PO-T2T, Ir(ppy)_3_ and 13PXZB. PO-T2T and Ir(ppy)_3_ were directly purchased from commercial sources, and 13PXZB was newly designed and synthesized as shown in Supporting Information. The cyclic voltammograms of the three materials are shown in Figure S1. Both Ir(ppy)_3_ and 13PXZB show nearly identical oxidation onsets, and their HOMO energy levels are accordingly estimated to be identically at −5.30 eV, while the LUMO energy level of PO-T2T is estimated to be −3.26 eV from the onset of the reduction curve. Combining the energy gaps determined from the onsets of absorption spectra, the LUMO energy levels are estimated to be −2.76 eV for Ir(ppy)_3_ and −1.95 eV for 13PXZB, and the HOMO energy level of PO-T2T is −6.93 eV. Based on our previous study, the driving force for exciplex formation can be approximately described as Equation (1) (Liu et al., 2015a)

(1) $$\begin{array}{r} -\Delta G_{\text{EX}}=HOMO_{\text{D}}-HOMO_{\text{A}}\left(for\;acceptor\right)\;or\;\\ -\Delta G_{\text{EX}}=LUMO_{\text{D}}-LUMO_{\text{A}}\left(for\;donor\right) \end{array}$$

Thus, the driving forces are approximately estimated to be 1.63 eV for PO-T2T and 0.5 eV for Ir(ppy)_3_ in the PO-T2T:Ir(ppy)_3_ system and 1.63 eV for PO-T2T and 1.31 eV for 13PXZB in the PO-T2T:13PXZB system. These high values can ensure the exciplex formation in both systems. Moreover, as the CT transition of exciplex happens between LUMO of A and HOMO of D, the exciplex energy can be described as Equation (2), where the constant is exciton binding energy and ranges from 0 to 0.20 eV (Kolosov et al., 2002)

(2) $$\begin{array}{r} E_{\text{exciplex}}=\text{e}\left(LUMO_{\text{A}}-HOMO_{\text{D}}\right)+constant \end{array}$$

Thus, both exciplexes possess energy of 2.02 eV approximately, ensuring they exhibit red emission. Moreover, the respective T_1_ energy levels of PO-T2T, Ir(ppy)_3_ and 13PXZB are 2.95, 2.45, and 2.73 eV, which are determined from the highest energy vibronic sub-band of their phosphorescence spectra at 77 K (Figure S2). These T_1_ energy values of components are much higher than both exciplex energies, which boosts the exciplex to harvest all excitons.

The absorption and photoluminescence (PL) spectra of PO-T2T:Ir(ppy)_3_ and PO-T2T:13PXZB exciplexes were investigated first. As shown in Figures 2B,C, the absorption spectra of both PO-T2T:Ir(ppy)_3_ and PO-T2T:13PXZB mixed films are nearly identical to their constituting molecules at room temperature, suggesting that there are no extra transitions generated in the ground states. Correspondingly, their PL spectra show broad emissions in the range of 480–733 nm with a peak at 590 nm for PO-T2T:Ir(ppy)_3_ and in the range from 480 to 670 nm with a peak at 562 nm for PO-T2T:13PXZB, which significantly differ from the PL spectra of the individual constituting molecules, proving the formation of exciplexes for both films during the photoexcitations. And the PL spectrum of PO-T2T:Ir(ppy)_3_ is slightly red-shifted compared with that of PO-T2T:13PXZB, indicating PO-T2T:13PXZB actually has higher energy than PO-T2T:Ir(ppy)_3_. Particularly, in the PL spectrum of PO-T2T:Ir(ppy)_3_ mixed film, a slight shoulder can be observed at the emission area of Ir(ppy)_3_, suggesting that the original metal-to-ligand CT transition of Ir(ppy)_3_ is still competitive during the photoexcitation.

**Figure 3:**
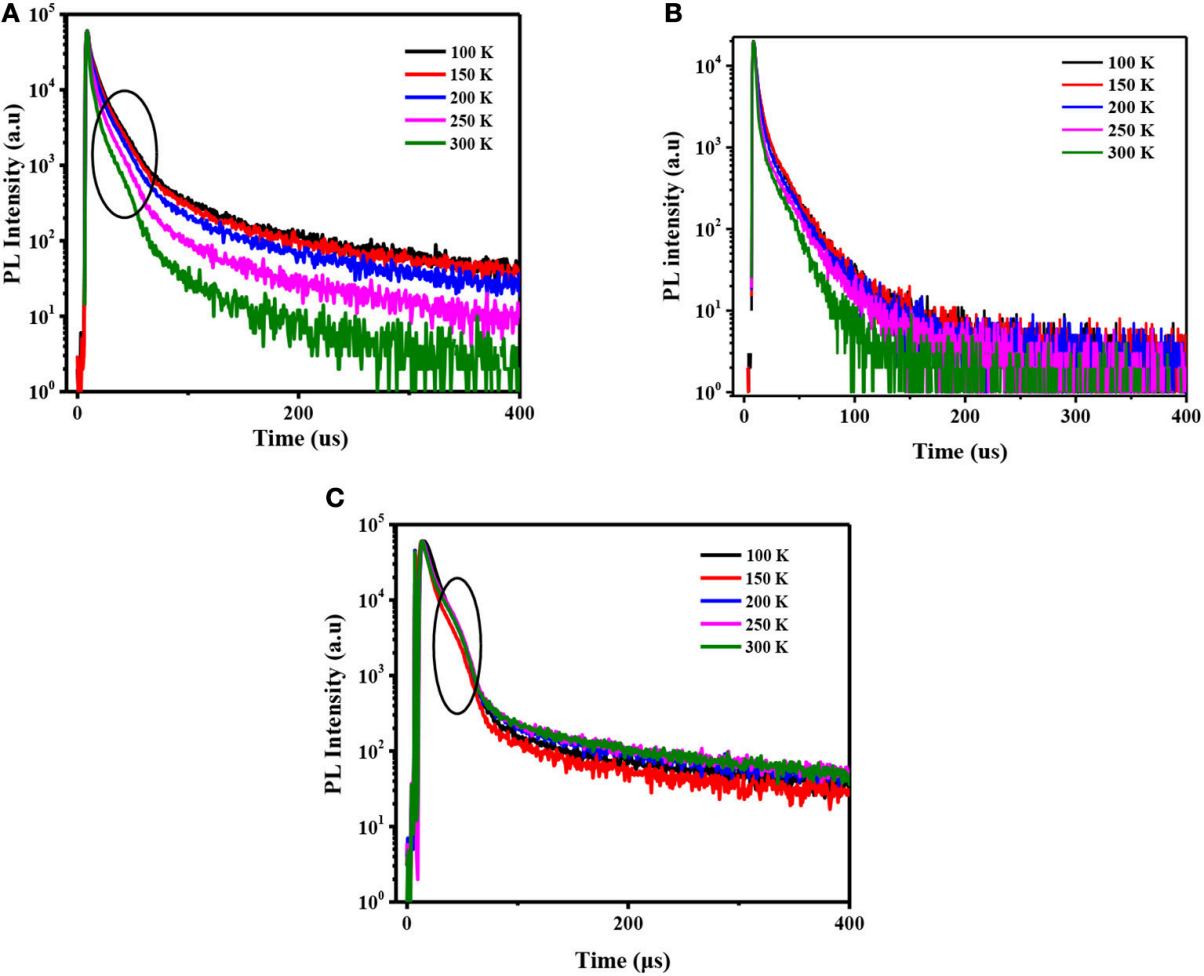
Transient PL decay curves of the **(A)** PO-T2T:Ir(ppy)_3_ film; **(B)** PO-T2T:13PXZB film; and **(C)** mCP:Ir(ppy)_3_ film at various temperatures by exciting at 300 nm.

To study the photophysical properties of both exciplexes, we further measured the fluorescence and phosphorescence spectra of the two mixed films at 77 K. As shown in Figures 2D,E, from the peaks in the fluorescence and phosphorescence spectra, the S_1_ and T_1_ energy levels of both the exciplex systems were estimated to be 2.162 and 2.136 eV for PO-T2T:Ir(ppy)_3_ and 2.215 and 2.188 eV for PO-T2T:13PXZB, respectively. Thus, their Δ*E*_ST_s are calculated to be 0.026 and 0.030 eV for PO-T2T:Ir(ppy)_3_ and PO-T2T:13PXZB, respectively. These extremely small Δ*E*_*ST*_s can lead to efficient RISC process from T_1_ to S_1_ state, which endow both exciplexes with TADF characteristic. Temperature-dependent transient decay characteristics of these two exciplexes were further measured under nitrogen atmosphere. As shown in Figure 3 and Figure S3, by exciting both the components at 300 nm, the lifetimes of both PO-T2T:Ir(ppy)_3_ and PO-T2T:13PXZB exciplexes show significantly decline along with the temperature increasing from 100 to 300 K, indicating their TADF characteristics. At room temperature, PO-T2T:Ir(ppy)_3_ shows a prompt lifetime of 13.2 ns and extremely small delayed lifetime of 2.8 μs. PO-T2T:13PXZB exciplex has a prompt lifetime of 17.1 ns and a decay lifetime of 13.9 μs, which is significantly longer than that of PO-T2T:Ir(ppy)_3_ film. This phenomenon is caused by the SOC effect of heavy metal core in phosphor, which can effectively enhance the RISC process. Moreover, in the range of <100 μs (shown in Figures 3A,C), significant turning curves can be observed with a similar behavior compared to the initial curve of Ir(ppy)_3_, which should indicate the evident contribution of exciplex phosphorescence. PL quantum yields (PLQYs) of PO-T2T:Ir(ppy)_3_ and PO-T2T:13PXZB mixed films with a thin thickness of 5 nm were measured via integrating sphere measurements in atmosphere. Both films present a similar low PLQY value of about 4%. Considering that most of triplet excitons can be quenched by oxygen, the PLQY value will be mainly contributed by the prompt component of singlet excitons, and both of the exciplexes should have similar luminescence efficiencies in the devices if we neglect the effect of triplet excitons (Méhes et al., 2012). While under oxygen-free condition, both singlet and triplet excitons make contributions to the emission. As a result, the PLQY values were increased to 23.3 and 8.6% for PO-T2T:Ir(ppy)_3_ and PO-T2T:13PXZB, respectively at room temperature. The evidently higher PLQY of PO-T2T:Ir(ppy)_3_ should be ascribed to the reduced NR decays of excited states. To further understand this point, PL spectra and delayed transient PL decays at various temperatures were measured and shown in Figure S4, and the data are extracted and summarized in Table S1. Different from conventional TADF emitters, the PL intensities are decreased from 200 to 300 K for both exciplexes. This result is because NR decays are significantly enhanced with the increased temperatures. The triplet formation efficiency (Φ_T_) and Δ*E*_ST_ were derived using a Berberan-Santos plot from the temperature-dependent results (in the Supporting Information) (Berberan-Santos and Garcia, 1996; Wang H. et al., 2014). The intersystem crossing rate constant (*k*_ISC_), the non-radiative rate of singlet excitons constants ($k_{\text{nr}}^{\text{s}}$) and the non-radiative of triplet excitons rate constant ($k_{\text{nr}}^{\text{T}}$) were also calculated assuming that *k*_ISC_ was independent of temperature, and summarized in Table S2. The *k*_ISC_ of PO-T2T:Ir(ppy)_3_ and PO-T2T:13PXZB were calculated to be 6.70 × 10^7^ and 4.93 × 10^7^ s^−1^. Meanwhile, the $k_{\text{nr}}^{\text{S}}$ of PO-T2T:Ir(ppy)_3_ and PO-T2T:13PXZB were calculated to be 0.73 × 10^7^ and 0.85 × 10^7^ s^−1^.Moreover, the $k_{\text{nr}}^{\text{T}}$ of the PO-T2T:Ir(ppy)_3_ and PO-T2T:13PXZB were estimated to 7.00 × 10^3^ and 1.04 × 10^4^ s^−1^, respectively. Obviously, the $k_{\text{nr}}^{\text{S}}$ and $k_{\text{nr}}^{\text{T}}$ of PO-T2T:Ir(ppy)_3_ are lower than the PO-T2T:13PXZB, proving the NR decays of S_1_ and T_1_ states are suppressed in phosphor-based exciplexes.

**Figure 4:**
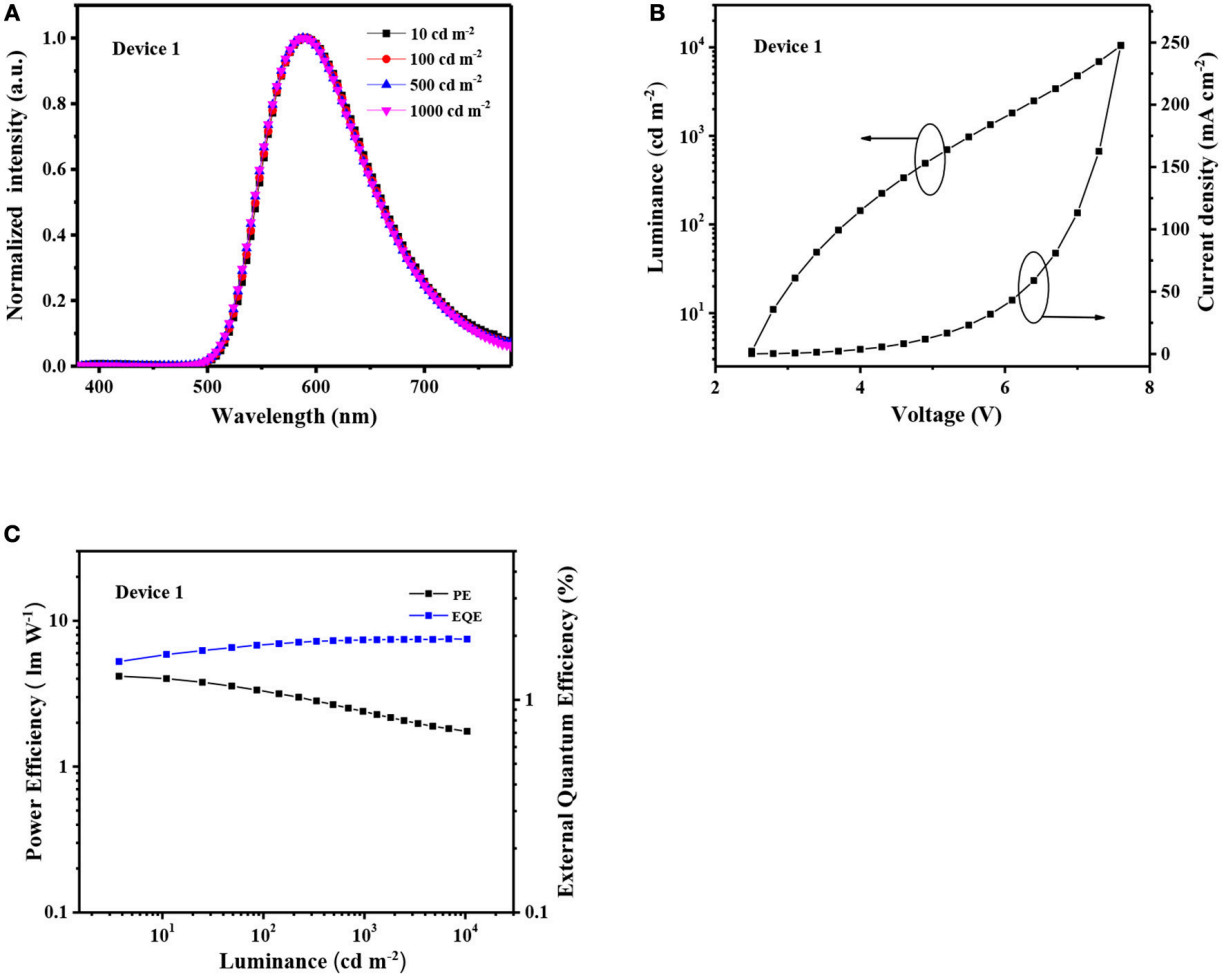
**(A)** EL spectra of the Device 1 at different luminance; **(B)** Current density–luminance-voltage characteristics of Device 1; **(C)** PE–EQE-luminance plots of the Device 1.

To evaluate the electroluminescence (EL) performance of the two exciplex, both PO-T2T:Ir(ppy)_3_ and PO-T2T:13PXZB exciplexes were used as the emitters to fabricate the devices, respectively. Due to the excellent electron transporting property of PO-T2T and good hole transporting property of 13PXZB (as shown in Figure S5), the PO-T2T:13PXZB-based device is constructed with a structure of ITO/TAPC (35 nm)/13PXZB (10 nm)/PO-T2T:x wt% 13PXZB (30 nm)/PO-T2T (45 nm)/LiF (1 nm)/Al (100 nm) (Device 1). In the device, cyclohexylidenebis[*N*,*N*-bis(4-methylphenyl)aniline] (TAPC) and PO-T2T are respectively used as the hole-transporting layer (HTL) and electron-transporting layer, ITO (indium tin oxide) and LiF/Al work as the anode and the cathode, respectively. A thin layer of 10 nm 13PXZB is inserted between HTL and the emitting layer (EML) aiming to avoid additional exciplex formed between TAPC and PO-T2T. The doping concentration of 13PXZB in EML is optimized to 40 wt% for Device 1. As shown in Figure 4, Device 1 exhibits a low turn-on voltage (at the brightness of 1 cd m^−2^) of 2.5 V and stable red EL emission at different luminances with a peak at 592 nm and a CIE coordinate of (0.52, 0.47), indicating that we have successfully constructed a red exciplex with a conventional material system of PO-T2T and 13PXZB. However, the maximum current efficiency (CE), power efficiency (PE), and EQE of Device 1 are only 4.2 cd A^−1^, 2.0 lm W^−1^ and 1.9%, respectively. Such low device efficiency should be ascribed to the evident NR decays of S_1_ and T_1_ states for conventional red exciplexes.

**Figure 5:**
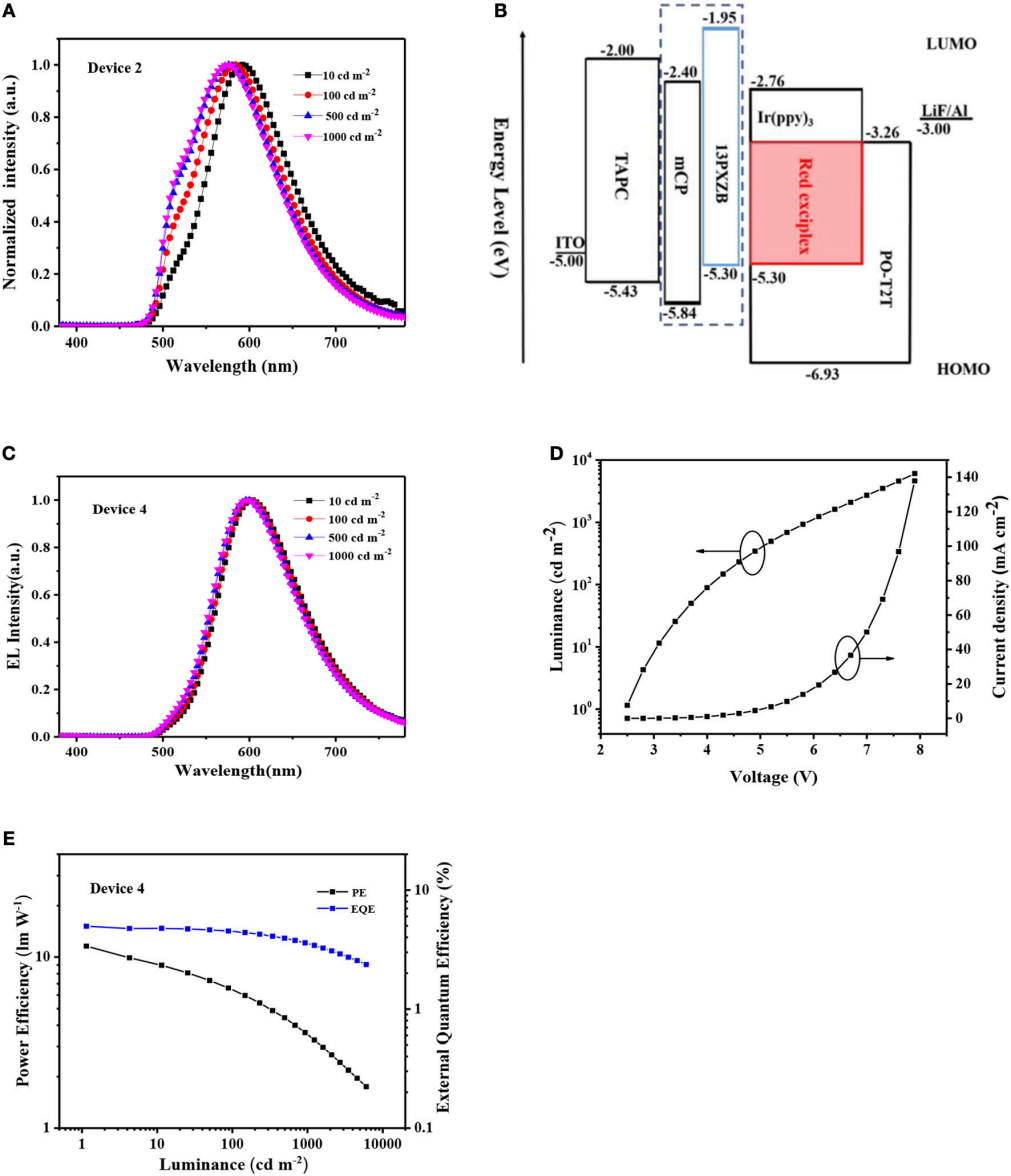
**(A)** EL spectra of the Device 2 at different luminance; **(B)** Device structures and the energy diagrams of Device 2 and 4; **(C)** EL spectra of the Device 4 at different luminance; **(D)** Current density–luminance-voltage characteristics of Device 4; **(E)** PE–EQE-luminance plots of the Device 4.

Different from PO-T2T:13PXZB exciplex, both PO-T2T and Ir(ppy)_3_ are electron transporting materials. As shown in Figure S5, the electron transporting capacity of PO-T2T:Ir(ppy)_3_ is significantly better than its hole transporting capacity. Thus, an electron-blocking layer (EBL) is needed to benefit carrier recombination in the device. We first constructed Device 2 with a structure of ITO/TAPC (35 nm)/mCP (10 nm)/PO-T2T:x wt% Ir(ppy)_3_ (30 nm)/PO-T2T (45 nm)/LiF (1 nm)/Al (100 nm). 3-bis(9H-carbazol-9-yl)benzene (mCP) is used as EBL due to its unipolar hole transporting capacity. And the doping concentration of Ir(ppy)_3_ is optimized to 8 wt%. However, as shown in Figure 5A, Device 2 exhibits unsatisfactory EL spectra with additional green emission around 520 nm which should be attributed to the phosphorescence of the initial Ir(ppy)_3_. This phenomenon is consistent with the PL spectrum as shown in Figure 2B, indicating competition of exciton harvest between Ir(ppy)_3_ and PO-T2T:Ir(ppy)_3_. In Device 2, the excitons are generated at the interface of mCP/PO-T2T:Ir(ppy)_3_. Beyond PO-T2T:Ir(ppy)_3_, the excited states of mCP:PO-T2T exciplex is also unavoidably generated. The energy of mCP:PO-T2T exciplex is around 2.63 eV (Liu et al., 2016), higher than the energies of Ir(ppy)_3_ and PO-T2T:Ir(ppy)_3_. The competition between Ir(ppy)_3_ and PO-T2T:Ir(ppy)_3_ should be leaded by the energy transfers from mCP:PO-T2T to Ir(ppy)_3_ and PO-T2T:Ir(ppy)_3_.

To avoid the harmful green emission, the exciton harvest of Ir(ppy)_3_ should be suppressed. Thus, we designed an optimized a device structure by changing mCP EBL to 13PXZB. At the 13PXZB/PO-T2T:Ir(ppy)_3_ interface, the excited states of PO-T2T:13PXZB can also generate. But the energy of PO-T2T:13PXZB is in between that of Ir(ppy)_3_ and PO-T2T:Ir(ppy)_3_, which can prevent the exciton harvest of Ir(ppy)_3and_ thus suppress the green emission. To further prove this point, we also fabricated a device with a structure of ITO/TAPC (35 nm)/13PXZB (10 nm)/PO-T2T (75 nm)/LiF (1 nm)/Al (100 nm) (Device 3). As shown in Figure S6, Device 3 exhibits the same EL spectra compared with Device 1 and slightly lower maximum efficiencies of 3.5 cd A^−1^ for CE, 2.9 lm W^−1^ for PE and 1.6% for EQE. The reason is that Ir(ppy)_3_ cannot harvest the excitons from the red exciplex PO-T2T:13PXZB.

Thus, for PO-T2T:Ir(ppy)_3_ exciplex, Device 4 was finally constructed with a structure of ITO/TAPC (35 nm)/13PXZB (10 nm)/PO-T2T:x wt% Ir(ppy)_3_ (30 nm)/PO-T2T (45 nm)/LiF (1 nm)/Al (100 nm). And the optimized doping concentration of Ir(ppy)_3_ in EML is also 8 wt%. As shown in Figure 5B, compared with mCP, 13PXZB also has more appropriate HOMO and LUMO energy levels, which can benefit the hole injection to EML and prevent the electron escape from EML. As shown in Figure 5C, a stable red EL emission with a peak at 604 nm and a CIE coordinate of (0.55, 0.44) is successfully generated, and the green emission from Ir(ppy)_3_ nearly disappeared in the spectra, indicating the feasibility of our device optimization. As shown in Figure S7, the EL spectrum of Device 4 is clearly red-shifted compared with that of Device 1 and 3. Such results indicate that not only the emission is from PO-T2T:Ir(ppy)_3_ exciplex, but also effective energy transfer is proved from PO-T2T:13PXZB to PO-T2T:Ir(ppy)_3_. As listed in Table 1 and shown in Figures 5C–E, Device 4 realizes a low turn-on voltage of 2.5 V and maximum CE, PE and EQE of 9.3 cd A^−1^, 11.6 lm W^−1^ and 5%, respectively. Such high EQE is 2.6 times and 3.1 times higher than that of Device 1 and 3, respectively. And EQE value of 5% is the highest result for red TADF OLEDs based on exiplex emitters. Considering both PO-T2T:13PXZB and PO-T2T:Ir(ppy)_3_ possess the same PLQY value of about 4% under triplet excitons quenched condition, the evident efficiency difference between Device 1, 3, and 4 indicates much higher triplet exciton utilization of PO-T2T:Ir(ppy)_3_, which should be ascribed to the beneficial effect of heavy metal ion core in the phosphor component. The SOC effect of heavy metal ion core can not only effectively suppress the NR decays of excited states for red TADF exciplexes, but also induce phosphorescence and make great contribution to EL emission. Our study provides a novel approach to develop efficient red TADF exciplexes with phosphors.

**Table 1:** Summary of performances of the exciplex-based devices.

**Device**	**V_on_ [V]^a^**	**λ_MAX_ [nm]**	**CE/PE/EQE^b^ [cd A^−1^/lm W^−1^/%]**			**CIE^c^ [x, y]**
			**Maximum**	**@ 100 cd m^**−2**^**	**@ 1,000 cd m^**−2**^**	
1	2.5	592	4.2/2.0/1.9	3.9/3.4/1.8	4.2/2.4/1.9	(0.52,0.47)
2	2.6	584	21.6/19.4/9.6	21.3/17.6/9.5	16.1/10.1/7.2	(0.48,0.49)
3	2.6	592	3.5/2.9/1.6	3.4/2.3/1.5	3.0/1.3/1.4	(0.52,0.47)
4	2.5	604	9.3/11.6/5.0	8.4/6.6/4.5	6.7/3.6/3.6	(0.55,0.44)

a *Turn-on voltage, estimated at 1 cd m^−2^*;

b *CE, current efficiency; PE, power efficiency; EQE, external quantum efficiency*;

c *Estimated at 100 cd m^−2^*.

## Conclusion

In summary, we present a novel strategy to construct efficient red TADF exciplexes by introducing phosphor as one component. The SOC effect of heavy metal ion core in phosphor can suppressed the NR decays of excited states and induced phosphorescence makes great contribution to total emission, thus improving the exciton utilization. Red TADF exciplex PO-T2T:Ir(ppy)_3_ is constructed accordingly, which exhibits high maximum efficiencies of 9.3 cd A^−1^ CE, 11.6 lm W^−1^ PE, and 5% EQE in the device. Such high EQE is 2.6 times higher than that of the device based on the comparative conventional red TADF exciplex PO-T2T:13PXZB and the best performance among reported red TADF OLEDs based on exciplex emitters. These results not only provide a new pathway to develop efficient exciplex emitters with vast phosphors, but also demonstrate the superiority of phosphor-based exciplexes.

## Author Contributions

C-JZ and X-HZ designed whole work. MZ, KW, and Y-ZS characterize the physical properties of compounds. MZ, KW, and HL fabricated and optimized the devices. D-QW and XL synthesized the new organic compound. MZ and KW wrote the paper with support from C-JZ, S-LT, and X-HZ. All authors contributed to the general discussion.

### Conflict of Interest Statement

The authors declare that the research was conducted in the absence of any commercial or financial relationships that could be construed as a potential conflict of interest.
