# Supplementary material for: Development of Red Exciplex for Efficient OLEDs by Employing a Phosphor as a Component
Source: Front Chem. 2019 Jan 31;7:16. doi: 10.3389/fchem.2019.00016 (PMC6365432; doi:10.3389/fchem.2019.00016)
Supplement: Supplementary file 1 [file Data_Sheet_1.docx]

Supporting Information

Development of Red Exciplex for Efficient OLEDs by Employing a Phosphor as a Component

*Ming Zhang, Kai Wang, Cai-Jun Zheng,* De-Qi Wang, Yi-Zhong Shi, Hui Lin, Si-Lu Tao, Xing Li, and Xiao-Hong Zhang**

1. **Synthesis.**

**1,3-di(10H-phenoxazin-10-yl)benzene (13PXZB)**.

Toluene (20 ml) and tri-tert-butyl phosphine solution 10% in pentane (0.3 mL, 0.13 mmol) were added to a mixture of phenoxazine (403.1mg, 2.20 mmol), 1,3-dibromobenzene (235.9 mg, 1.00 mmol), palladium(II) acetate (6.7 mg, 0.03 mmol), sodium tertbutoxide (576.63 g, 6.00mmol). With stirring, the suspension was heated at 90°C for 12 h under nitrogen atmosphere. When cooled to room temperature, the mixture was extracted with dichloromethane and dried over MgSO_4_. After the solvent had been removed, the residue was purified by column chromatography on silica gel using dichloromethane as the eluent to give a white solid, with an 83.6% yield (368 mg). ^1^H NMR (600 MHz, CDCl_3_) δ 7.82 (t, J = 7.9 Hz, 1H), 7.48 (dd, J = 7.9, 2.0 Hz, 2H), 7.40-7.39 (m, 1H), 6.69 (td, J = 7.8, 1.7 Hz, 5H), 6.66 (dd, J = 7.7, 1.7 Hz, 3H), 6.63 (td, J = 7.6, 1.8 Hz, 4H), 5.99 (dd, J = 7.8, 1.6 Hz, 4H). TOF-MS (EI) m/z: [M]^+^: calcd for C_30_H_20_N_2_O_2_ 440.15; found, 440.15.

**2. Experimental section**

**2.1** **Materials sources.** All materials used were directly purchased from directly purchased from commercial sources, except 13PXZB which is newly designed and synthesized.

**2.2 General information.** ^1^H NMR data and mass spectral data were respectively measured with Bruker Advance-400 spectrometer and Finnigan 4021C gas chromatography-mass spectrometry instrument. The measurements of fluorescence quantum yields (PLQY), elemental analysis, and PL temperature-dependent transient decay lifetime were carried out at the Analysis and Test Center of Soochow University of Materials Chemistry institute. Cyclic voltammetry data were measured with CHI660E electrochemical analyzer, with a saturated calomel electrode (SCE), Pt disks, and 0.1M Bu_4_NPF_6_ respectively as the reference electrode, working electrode, and the supporting electrolyte, and the scan rate of 10 mV/s.

**2.3** **OLEDs fabrication.** The ITO coated glasses were cleaned and treated with UV-ozone for 15 minutes, and then organic materials were deposited in a vapor deposited instrument under the pressure of 4 × 10^–4^ Pa. The deposited rate of organic materials, LiF, and Al were controlled at 1-2 Å s^–1^, 0.1 Å s^–1^, and 10 Å s^–1^, respectively. The optical and electrical data of the devices were respectively recorded with a PR650 Spectrascan and Keithley 2400 SourceMeter under ambient atmosphere simultaneously. CE, PE, and EQE were calculated with the data of current, luminance and emission spectrum, assuming a Lambertian distribution.

**3. Determination of Φ_p_ ,Φ_d_ and *k*_ISC._**

In this study, total PL quantum efficiency was determined by the emission intensity between in air (I_air_) and in vacuum (I_vacuum_) and Φ_total_ was calculated by the flowing equation:

$\Phi_{\text{total}}=\Phi_{\text{air}}\frac{\text{I}_{\text{vacuum}}}{\text{I}_{\text{air}}}$ (1)

The Φ_prompt_ and Φ_delayed_ were determined by using total PL quantum efficiency and the ratio between prompt and delayed components which was calculated from transient PL measurements. The intensity ratio between prompt (r_1_) and delayed (r_2_) components were determined using emission lifetime (τ_1_, τ_2_) and fitting parameter (Α_1_, Α_2_) as follow.

$I(\mathrm{t})=\mathrm{A}_{1}e^{-\frac{t}{\tau_{1}}}+\mathrm{A}_{2}e^{-\frac{t}{\tau_{2}}}$ (2)

$r_{1}=\frac{\text{A}_{1}\tau_{1}}{\text{A}_{1}\tau_{1}\text{+A}_{2}\tau_{2}}$ (3)

$r_{2}=\frac{\text{A}_{2}\tau_{2}}{\text{A}_{1}\tau_{1}\text{+A}_{2}\tau_{2}}$ (4)

Then, Φ_prompt_ and Φ_delayed_ were determined using intensity ratio (r_1_, r_2_) and total emission quantum yield.

$\ln\left[ \frac{I_{prompt}}{I_{delayed}}-\left( \frac{1}{\Phi_{T}}-1 \right) \right]=ln\left\{ \frac{k_{p}+k_{nr}^{T}}{k_{RISC}} \right\}+\frac{{\Delta E}_{ST}}{RT}$(5)

The energy difference (Δ*E*_ST_) and the triplet formation efficiency (Φ_T_) were evaluated using a Berberan-Santos plot^[S2]^ from the temperature dependence results according to the above equation. Then, the intersystem crossing rate constant (*k*_ISC_), were estimated assuming that *k*_ISC_ was independent of temperature

**3.1. Main kinetic parameters calculations.**

According to the literature, the main kinetic parameters of the two exciplexs were calculated by the following equations. Particularly, to conveniently evaluate the phosphor-based exciplexes, we neglect the phosphorescence decay.

$\text{k}_{\text{p}}\text{=}1/\tau_{\text{p}}$ (6)

$\text{k}_{\text{d}}\text{=}1/\tau_{\text{d}}$ (7)

$\text{k}_{\text{r}}^{\text{S}}\text{=k}_{\text{p}}\Phi_{\text{F}}$ (8)

$\text{k}_{\text{ISC}}\text{=k}_{\text{p}}\Phi_{\text{ISC}}$ (9)

$\text{k}_{\text{p}}\text{=k}_{\text{r}}^{\text{S}}\text{+k}_{\text{nr}}^{\text{S}}\text{+k}_{\text{ISC}}$ (10)

$\text{k}_{\text{RISC}}\text{=}\frac{\text{k}_{\text{p}}\text{k}_{\text{d}}}{\text{k}_{\text{ISC}}}\frac{\Phi_{\text{TADF}}}{\Phi_{\text{F}}}$ (11)

$\text{k}_{\text{nr}}^{\text{T}}\text{=k}_{\text{d}}\text{-(1-}\frac{\text{k}_{\text{ISC}}}{\text{k}_{\text{r}}^{\text{S}}\text{+k}_{\text{nr}}^{\text{S}}\text{+k}_{\text{ISC}}}\text{)k}_{\text{RISC}}$ (12)

**Figure S1.** Cyclic voltammetry of a) 13PXZB, Ir(ppy)_3_ and PO-T2T in N,N-dimethylformamide (DMF); and b) mCP in acetonitrile (ACE). The HOMO levels of 13PXZB and Ir(ppy)_3_, LUMO level of PO-T2T (in DMF) are respectively calculated with equation: HOMO = − *e*(*E*_ox_ + 4.52) and LUMO = − *e*(*E*_red_ + 4.52). The HOMO level of mCP (in ACE) is calculated with equation: HOMO = − *e*(*E*_ox_ + 4.62). *E*_ox_, *E*_red_ are the onsets of oxidation and reduction curves, respectively.

**Figure S2.** Phosphorescence spectra of a) PO-T2T, b) Ir(ppy)_3_ and c) 13PXZB in 2-MeTHF at 77K.

**Figure S3.** Transient PL decay curves of the (a) PO-T2T:Ir(ppy)_3_ film; (b) PO-T2T:13PXZB film in the range of 100 ns at room temperatures by exciting at 300 nm.

**Figure S4. a)** PL spectra of PO-T2T:Ir(ppy)_3_ film at various temperature; b) PL spectra of PO-T2T:13PXZB film at various temperature; c) the transient PL decay curves of PO-T2T:Ir(ppy)_3_ film at various temperature; d) the transient PL decay curves of PO-T2T:13PXZB film at various temperature.

**Table S1.** Lifetime data extracted from the transient characterization of PO-T2T:Ir(ppy)_3_ film and PO-T2T:13PXZB film in different temperature.

| exciplex | T(k) | τ_1_ (ns) | A_1_ | τ_2_(μs) | A_2_ | Φtotal | Φ_p_ | Φ_d_ |
| --- | --- | --- | --- | --- | --- | --- | --- | --- |
| PO-T2T:Ir(ppy)_3_ | 300 | 13.1 | 0.491 | 2.8 | 0.392 | 0.233 | 0.023 | 0.210 |
|  | 280 | 13.5 | 0.511 | 4.1 | 0.376 | 0.332 | 0.036 | 0.296 |
|  | 260 | 13.9 | 0.701 | 5.6 | 0.312 | 0.389 | 0.047 | 0.342 |
|  | 240 | 14.7 | 0.739 | 7.3 | 0.284 | 0.445 | 0.062 | 0.383 |
|  | 220 | 15.3 | 0.796 | 8.2 | 0.257 | 0.501 | 0.075 | 0.426 |
|  | 200 | 16.1 | 0.766 | 11.3 | 0.242 | 0.565 | 0.096 | 0.469 |
| PO-T2T:13PXZB | 300 | 17.2 | 0.545 | 13.9 | 0.555 | 0.086 | 0.010 | 0.076 |
|  | 280 | 17.8 | 0.694 | 14.1 | 0.306 | 0.114 | 0.014 | 0.100 |
|  | 260 | 18.3 | 0.673 | 15.3 | 0.327 | 0.122 | 0.015 | 0.107 |
|  | 240 | 18.9 | 0.713 | 15.9 | 0.287 | 0.127 | 0.018 | 0.109 |
|  | 220 | 19.6 | 0.762 | 16.2 | 0.298 | 0.134 | 0.021 | 0.113 |
|  | 200 | 20.1 | 0.785 | 17.8 | 0.235 | 0.142 | 0.027 | 0.115 |

**Table S2**. Transition rates of two exciplex at 300 K. The unit of transition rates is s^-1^.

| Exciplex | Φ_total_ | Φ_prompt_ | Φ_TADF_ | τ_p_ (ns) | τ_d_ (μs) | k_p_ | K_d_ | K_r_^s^ | K_nr_^s^ | K_ISC_ | K_RISC_ | K_nr_^T^ |
| --- | --- | --- | --- | --- | --- | --- | --- | --- | --- | --- | --- | --- |
| PO-T2T:Ir(ppy)_3_ | 23.3 | 2.3 | 21.0 | 13.2 | 2.8 | 7.60×10^7^ | 3.57×10^5^ | 0.17×10^7^ | 0.73×10^7^ | 6.70×10^7^ | 3.71×10^6^ | 7.00×10^3^ |
| PO-T2T:13PXZB | 8.6 | 1.0 | 7.6 | 17.1 | 13.9 | 5.84×10^7^ | 7.21×10^4^ | 0.06×10^7^ | 0.85×10^7^ | 4.93×10^7^ | 6.17×10^5^ | 1.04×10^4^ |

 ****

**Figure S5.** Current density-voltage curves of hole- and electron-only devices based on a) 13PXZB; b) PO-T2T; c) PO-T2T:Ir(ppy)_3_. Hole-only devices: ITO/MoO_3_ (12 nm)/Materials (50 nm)/MoO_3_ (12 nm)/Al (100 nm); Electron-only devices: ITO/Al (40 nm)/LiF(1 nm)/Materials (50 nm)/LiF (1 nm)/Al (100 nm).

**Figure S6.** a) PE–EQE-luminance plots of the Device 2, b) Current density–luminance-voltage characteristics of Device 2; c) EL spectra of the Device 3 at different luminance; d) Current density–luminance-voltage characteristics of Device 3; c) PE–EQE-luminance plots of the Device 3.

**Figure S7.** EL spectra of the Device 1, 3 and 4.

**References**

[S1] T. Nakagawa, S.-Y. Ku, K.-T. Wong, C. Adachi, *Chem. Commun*. **2012**, *48*, 9580.

[S2] M. N. Berberan-Santos, J. M. M. Garcia, *J. Am. Chem. Soc.* **1996**, *118*, 9391.
